# Understanding physician antibiotic prescribing behavior for children with enterovirus infection

**DOI:** 10.1371/journal.pone.0202316

**Published:** 2018-09-07

**Authors:** Kuang-Che Kuo, Yi-Chun Yeh, Ying-Hsien Huang, I-Ling Chen, Chen-Hsiang Lee

**Affiliations:** 1 Department of Pediatrics, Kaohsiung Chang Gung Memorial Hospital and Chang Gung University College of Medicine, Kaohsiung, Taiwan; 2 Department of Psychiatry, Kaohsiung Medical University Hospital, Department of Psychiatry, Faculty of Medicine, Graduate Institute of Medicine, College of Medicine, Kaohsiung Medical University, Kaohsiung, Taiwan; 3 Department of Pharmacy, Kaohsiung Chang Gung Memorial Hospital and Chang Gung University College of Medicine, Kaohsiung, Taiwan; 4 Department of Internal Medicine, Kaohsiung Chang Gung Memorial Hospital and Chang Gung University College of Medicine, Kaohsiung, Taiwan; Center for Cell Biology (CBC/UA), University of Aveiro, PORTUGAL

## Abstract

**Background:**

Our previous study demonstrated that pediatricians prescribe antibiotics without proper clinical justification to patients with enterovirus infection, although antibiotics are not effective in treating the infections caused by these viruses. To improve the quality of healthcare, we aim to evaluate the association of clinical and demographic characteristics of patients and further to identify the determining factors for prescribing antibiotics to children experiencing enterovirus infection.

**Methods:**

We retrospectively reviewed the medical records of children who were hospitalized between January 2008 and December 2016 with a diagnosis of herpangina or hand-foot-mouth disease (HFMD). We identified those children who were prescribed antibiotics for at least 24 hours during admission. We conducted a retrospective descriptive study to analyze data in order to determine the factors associated with pediatrician antibiotics prescribing for enterovirus infection.

**Results:**

In the nine years of study period, the rate of antibiotics use was about 13% in these patients. A total of 3659 patients were enrolled during 2008~2012 and analyzed in detail. Elevated levels of C-reactive protein (CRP) and presence of leukocytosis in blood (WBC) were both significantly associated with pediatrician antibiotic prescribing for enterovirus infection (p<0.001). Between different specialistic devisions, there was significantly different proportion of antibiotics utilization for patients. In further analysis of antibiotics prescribing by Receiver operating characteristic (ROC) curve method, the level of CRP significantly had more the area under curve (0.708) compared with the count of WBC (p<0.05).

**Conclusions:**

The present study indicates that higher serum level of CRP is strongly associated with pediatricians prescribing antibiotics for children experiencing herpangina or HFMD. Antibiotic prescribing is a complex process. Pediatricians should be more judicious in decision-making time by their specialistics. Our findings would shed new light on process and allay the concern about inappropriate antibiotics.

## Introduction

Herpangina and hand-foot-mouth disease of enteroviral diseases, are prevalent at epidemic levels each year in Taiwan and Southeast Asia [1–4]. These viral infections impose a health and economic burden, causing suffering in children and accounting for large medical payments in healthcare. [5] Previous studies have focused on clinical manifestations and therapeutic strategies related to these serious enteroviral diseases. Due to implementation of these published therapeutic strategies in practice, such as intravenous immunoglobulin and extracorporeal membrane oxygenation, morbidity and mortality associated with enteroviruses have decreased over time.

In our previous study, we found that antibiotics were also utilized for these viral diseases with high CRP serum level, although they are not clinically indicated or effective[5]. Based on several previous studies, it is known that unnecessary antibiotic use has the potential to increase antimicrobial resistance and endanger the future effectiveness of antibiotics, as well as create the risk of the spread of antibiotic-resistant bacterial infections [6–8]. Concerns regarding inappropriate prescription in cases were demanded more detailed studies into physicians’ decision-making process about prescribing antibiotics. Therefore, several qualitative studies were focused on understanding the factors influencing physician antibiotic prescribing behavior; factors were found to be associated with physicians’ attitudes and knowledge [9–11]. Accordingly, the factors impacting prescribing of antibiotics were divided into intrinsic and extrinsic groups. In intrinsic groups, physician’s attitudes were associated with their complacency or fear. In extrinsic groups, patient-related factors (e.g. sign and symptoms) were the most commonly reported, and almost needed judgment by the professional background of physicians[10]. To our knowledge, the literature regarding the decision-making process of pediatricians in prescribing antibiotics is limited and there are fewer findings about the reasons for pediatricians making such decisions in caring for their patients.

In this study, our aim was to investigate retrospectively the extrinsic influencing factors (e.g. patients’ clinical and demographic characteristics) of antibiotic prescription by pediatricians for children experiencing herpangina or hand-foot-mouth disease. An improved understanding of the behavior of pediatricians in prescribing antibiotics will decrease abuse of antibiotics in patients. The findings of this study could generate more effective therapeutic strategies and increase the quality of healthcare for enteroviral infectious diseases.

## Materials and methods

### Study population and definition

We supposed that extrinsic influencing factors of antibiotics prescription behavior could be associated with pediatrician’s speciality and patient’s clinical presentations. This study was designed as retrospectively reviewing medical records of inpatients in a single medical center. In the first part, the study period was during the period from January 1, 2008 to December 31, 2012. Patients were selected by their diagnosis regarding to the episodes of admission. We identified infant and children (age<18 years old) experiencing herpangina (074.0) or HFMD (074.3), according to International Classification of Diseases Ninth Revision (ICD-9) code, from the inpatient database of the hospital as described in detail previously[5]. Because this study was a retrospective case-control design to find out possible extrinsic influencing factors, exploring the intrinsic factors by interviewing pediatricians was not done. Therefore, our main work was to carefully evaluate possible clues of patients on medical records, such as their clinical and demographic characteristics. The laboratory data in this study were obtained at the time of the first clinical evaluation of the patient during admission. The study definition of antibiotic therapy was a prescription of antibacterial agents during admission. The medical records of these cases were retrospectively reviewed for laboratory and demographic data, clinical outcomes, and antibiotic therapy regimens associated with each hospital stay. All date were fully available without restriction.

Discharges were assigned to diagnosis related groups (DRG), which are groups of patients with similar clinical presentation who are expected to require similar amounts of hospital resources. Each DRG has a relative weight that reflects the expected cost of inpatient treatment for patients in that group. The case mix index (CMI) is the mean of the DRG weight. DRGs provide not only the parameters of prospective payment on admission, but also provide information about the severity and outcome of disease [12–14]. We use the CMI to represent the severity of each inpatient in this study. By antibiotic prescribing, we divided all patients into two groups and compared the demographic and clinical manifestations between them.

For further understanding the antibiotics prescribing behavior of pediatricians, this study was trended to design as a retrospective case-control (1:2) study, by matching age, gender, and CMI to compare the influencing factors of antibiotic prescribing. The matching analysis was according to their propensity scores.

### Comparison with antibiotics prescribing behavior between the first period (2008~2012) and the second period (2013~2016)

For understanding pediatricians’ prescribing behavior without active education, we extended our study period until December 31, 2016 from our inpatient’s medical database according to the ICD-9 code (074.0 or 074.3) and ICD-10 code (B08.5 or B08.4). By the date of admission, patients were divided into two groups as the first period (year 2008~2012) and the second period (year 2013~2016). Enrolled cases were hospitalizing only in pediatric wards and on the service of pediatric divisions. Cases with missing laboratory data and demographic record, on surgeon service, and death were excluded. Other definition of variables were the same as the above-mentioned.

Ethical approval was received from the Chang Gung Medical Foundation Institutional Review Board, who approved this study as a retrospective analysis. Their IRB waived the need for consent of the medical records and each data was accessed anonymously.

### Statistical analysis

In descriptive statistics, categorical variables were expressed as frequency and proportion and continuous variables were summarized as mean ± standard error and range. By the normality of the distribution, either Student’s t tests (unpaired, two-tailed) or Mann-Whitney U tests were used for comparison of continuous variables between groups. A p-value <0.001 was considered statistically significant because our case number was large. Differences in frequency and proportion between groups were calculated using χ^2^ tests. Furthermore, all risk factors possibly associated with hospital stay were analyzed by linear regression. All risk factors possibly associated with antibiotics utilization were analyzed by logistic regression. The receiver operating characteristics (ROC) curve method was used to differentiate between the level of C-reactive protein (CRP) and the count of white blood cells (WBC); different cut-off points had different sensitivities and specificities for the prescription of antibiotics. The cut-off points for the prescription of antibiotics by WBC and CRP level were based on the highest value of sensitivity plus specificity identified by ROC curve. All statistical tests were performed by SPSS version 17.0 (SPSS Inc., Chicago, IL, USA).

Comparison of the ROC curves between CRP level and WBC were performed statistically by MedCalc version 12.7. The software calculates the difference between the areas under the ROC curves and displays standard error, 95% confidence intervals, and p-values. A p-value <0.05 was considered statistically significant.

## Results

This retrospective study was conducted at the Kaohsiung Chang Gung Memorial Hospital in southern Taiwan. It had 170 pediatric unit beds and 53 intensive care unit beds to provide primary and tertiary care for children. In our hospital, there are 10 divisions of pediatric department and pediatric surgery division to provide healthcare. In the first part of this study, the age range of participants was from 0 to 15 years old and their mean age was 2.13 years old. As described detail in our previously publishing report[5], during this period, 3659 episodes of infants and children admitted due to herpangina and HFMD were identified. For 3562 out of 3659 episodes, the laboratory data of CRP level and total WBC count were available. Laboratory data from the other 97 episodes were missing data and they were not included in the subsequent analysis. Antibiotics had been prescribed during 545 out of the 3659 (15%) admissions. However, our previous study mainly indicated that antibiotics could be neither appropriate nor beneficial in these patients with high CRP serum level[5]. Differently, this study was conducted to understand why pediatricians prescribed antibiotics for treatment of enteroviral diseases.

The clinical manifestations and demographic data of all enrolled cases were compared by antibiotic prescription status and are presented in Table 1. The cost of antibiotic consumption charged to Taiwanese healthcare insurance for treatment of the viral illness during the hospital stay was nearly US $7809.8. Penicillin analogues, including amoxicillin±clavunate (57%) were the most frequently prescribed, followed by the third generation of cephalosporin (29.5%). Length of hospital stay was found to be positively correlated with admission to the intensive care unit (ICU stay), antibiotic utilization, use of intravenous immunoglobulin (IVIG), and elevated CMI, but not with CRP levels, in multiple linear regression analysis (Table 2). These variables may represent more serious health conditions and higher severity enterovirus, and therefore be associated with a longer length of hospital stay. Our results revealed that higher CRP levels were not positively correlated with the severity of enteroviral diseases.

**Table 1:** Demographic and clinical characteristics of patients associated with the utilization of antibiotics.

Factors	Antibiotics	Yes	No	P value
		Case group N = 545	Control group N = 3114	
Sex (Male /Female)		313/232	1807/1307	0.794
Age (years) ^a^		2.34±0.10, (0~14)	2.10±0.04, (0~15)	0.040
Relative weight of DRG ^a^		0.269±0.005, (0.25~2.71)	0.267±0.000, (0.25~0.29)	0.000*
Cases on epidemic year				0.051
2008		145	898	
2009		93	440	
2010		170	1032	
2011		73	320	
2012		64	424	
CRP (mg/L)^a^		58.70±2.13, (0.2~276.6)	22.61±0.43, (0.2~224.7)	0.000*
WBC (x1000cell/mm^3^) ^a^		15.11±0.27, (3.0~43.9)	12.35±0.08, (2.3~36.2)	0.000*
ICU stay		20	74	0.078
Use of IVIG		14	31	0.002
Outcome				
Death		0	1	0.676
Stay in hospital (days)		4.39±0.09	3.62±0.03	0.000*

^a^: mean±SE, range

*: by Mann-Whitney test, significant statistically (p<0.001).

**Table 2:** Multiple linear regression analysis of factors associated with the length of hospital stay for children having herpangina/hand-foot-mouth disease.

Variable	Coefficients	S.E.	P value
Intercept	2.448	0.171	<0.001*
ICU stay (yes = 1, no = 0)	1.658	0.221	<0.001*
Antibiotics utilization (yes = 1, no = 0)	0.758	0.084	<0.001*
CMI	3.645	0.570	<0.001*
Use of IVIG (yes = 1, no = 0)	1.767	0.314	<0.001*
CRP (mg/L)	-0.004	0.001	<0.001*
WBC (x1000/mm^3^)	0.019	0.006	0.001

S.E.: standard error of coefficient.

Adjusted multiple R^2^ = 10.9%.

*: significant statistically (p<0.001).

The goodness-of-fit (p = 0.000).

**Fig 1:**
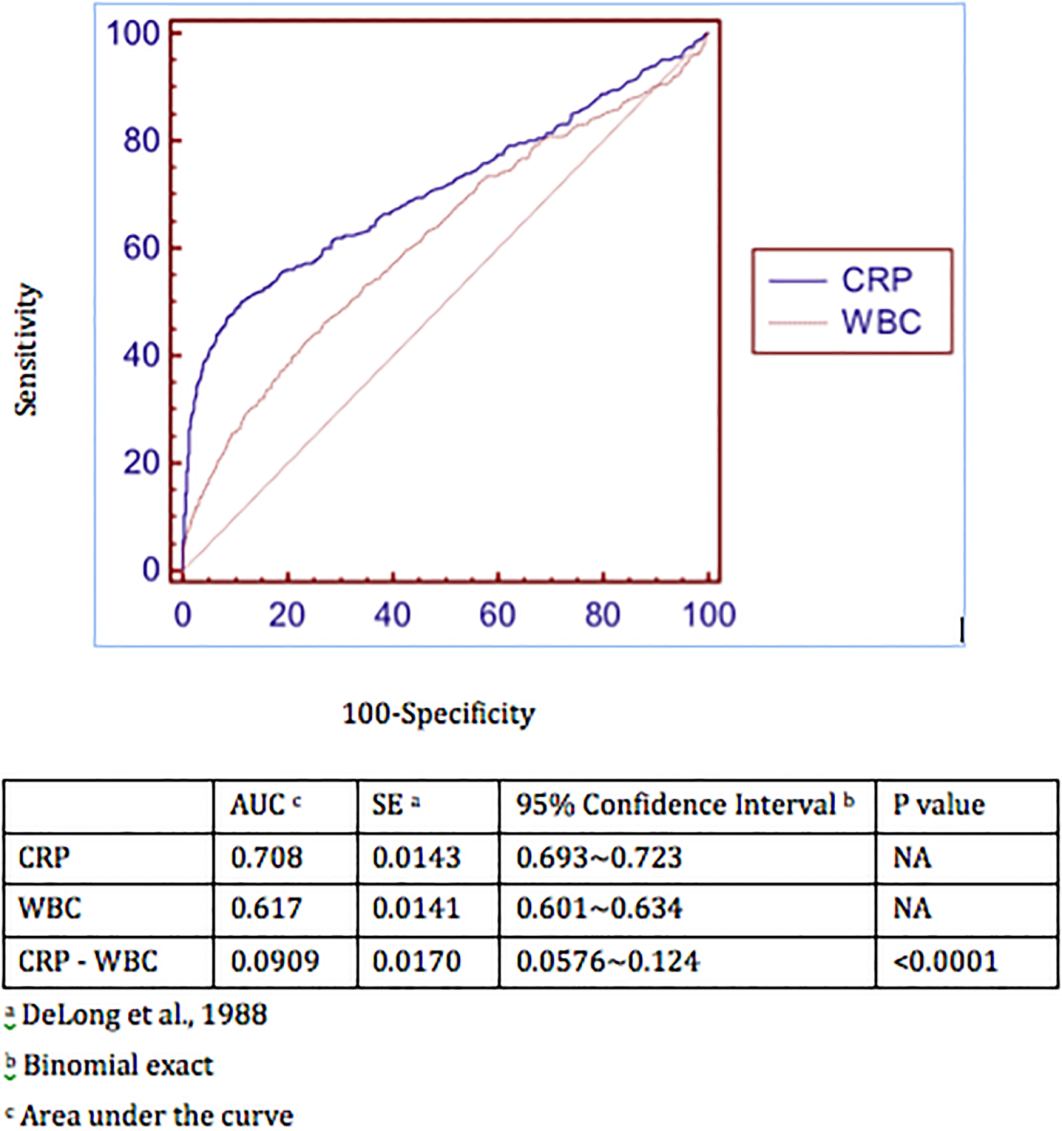
Comparison of ROC curves between CRP and WBC in blood of patients for the decision of antibiotics prescription.

Single variables were compared to analyze as risk factors for antibiotic prescribing (Table 1). The elevation of WBC and CRP level in blood, and disease severity of CMI were factors significantly associated with antibiotic prescription. In the multiple logistic regression analysis (Table 3), we found that these 2 variables of WBC and CRP level were still significantly associated with prescription of antibiotics in our patient population (p<0.001). Therefore, our findings indicate that these 2 factors play important roles in prescribing antibiotics clinically. In comparing the ROC curves between CRP level and WBC (Fig 1), the area under the curve (AUC) of CRP level was significantly more than that of WBC (p<0.05). This indicated that elevated CRP level was the strongest risk factor for prescription of antibiotics in children experiencing enterovirus infection. The cut-off level of serum CRP was 30 mg/L (sensitivity 62%; specificity 73%). In case-control (1:2) matching study, levels of CRP and WBC in these patients were still associated with antibiotic prescription in Table 4. However, the difference of specialistic division was significantly associated with antibiotics utilization (p<0.001).

**Table 3:** Multiple logistic regression analysis of factors associated with antibiotics utilization in children having herpangina/hand-foot-mouth disease.

Factor	Comparison	OR (95% C.I.)*	p value
CRP (mg/L)	per 1 unit increase	1.028 (1.024~1.031)	<0.001
WBC (x10^3^cell/mm3)	per 1 unit increase	1.076 (1.054~1.098)	<0.001
Age (year)	per 1 unit increase	1.067 (1.014~1.122)	0.013

*OR, odd ratio; C.I., confidence interval

Hosmer-Lemeshow goodness-of-fit (p = 0.61).

**Table 4:** Demographic and clinical characteristics of patients in matching case-control (1:2) study of the utilization of antibiotics by propensity score.

	Antibiotics Prescription		
Factors	Case group (yes)	Control group (no)	P value
	N = 543	N = 1086	
Age (y/o)^a^	2.34±0.09,(0~14)	2.35±0.07,(0~15)	0.884
Gender (M/F)	311/232	579/507	0.130
Weight of CMI ^a^	0.27±0.01,(0.25~2.71)	0.26±0.00,(0.25~0.29)	0.113
ICU admission (Y/N)	20/523	22/1064	0.047
IVIG use (Y/N)	14/529	8/1078	0.002
WBC (x 1000/mm^3^) ^a^	15.15±0.27,(3.0~43.9)	12.47±0.14,(2.3~33.6)	<0.001*
CRP (mg/L) ^a^	58.82±2.13,(0.2~276.6)	23.65±0.78,(0.2~224.7)	<0.001*
Length of hospital stay (days) ^a^	4.37±0.09,(1~20)	3.55±0.05,(1~11)	<0.001*
Death	0	0	NA

^a^: mean±SE, range.

*: by Mann-Whitney test, significant statistically (p<0.001).

**Fig 2:**
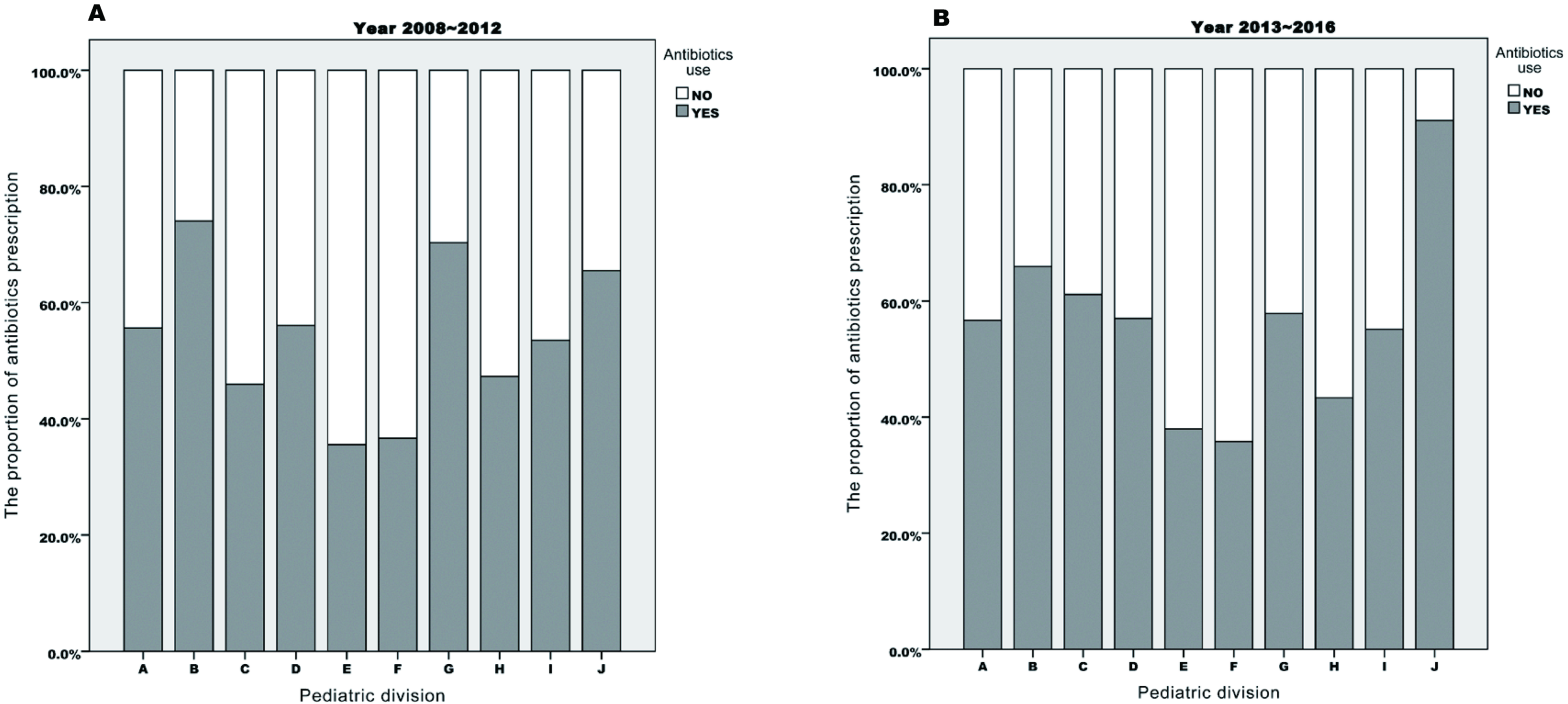
Comparison of antibiotics prescribing behavior between 10 divisions of Pediatrics.

**Fig 3:**
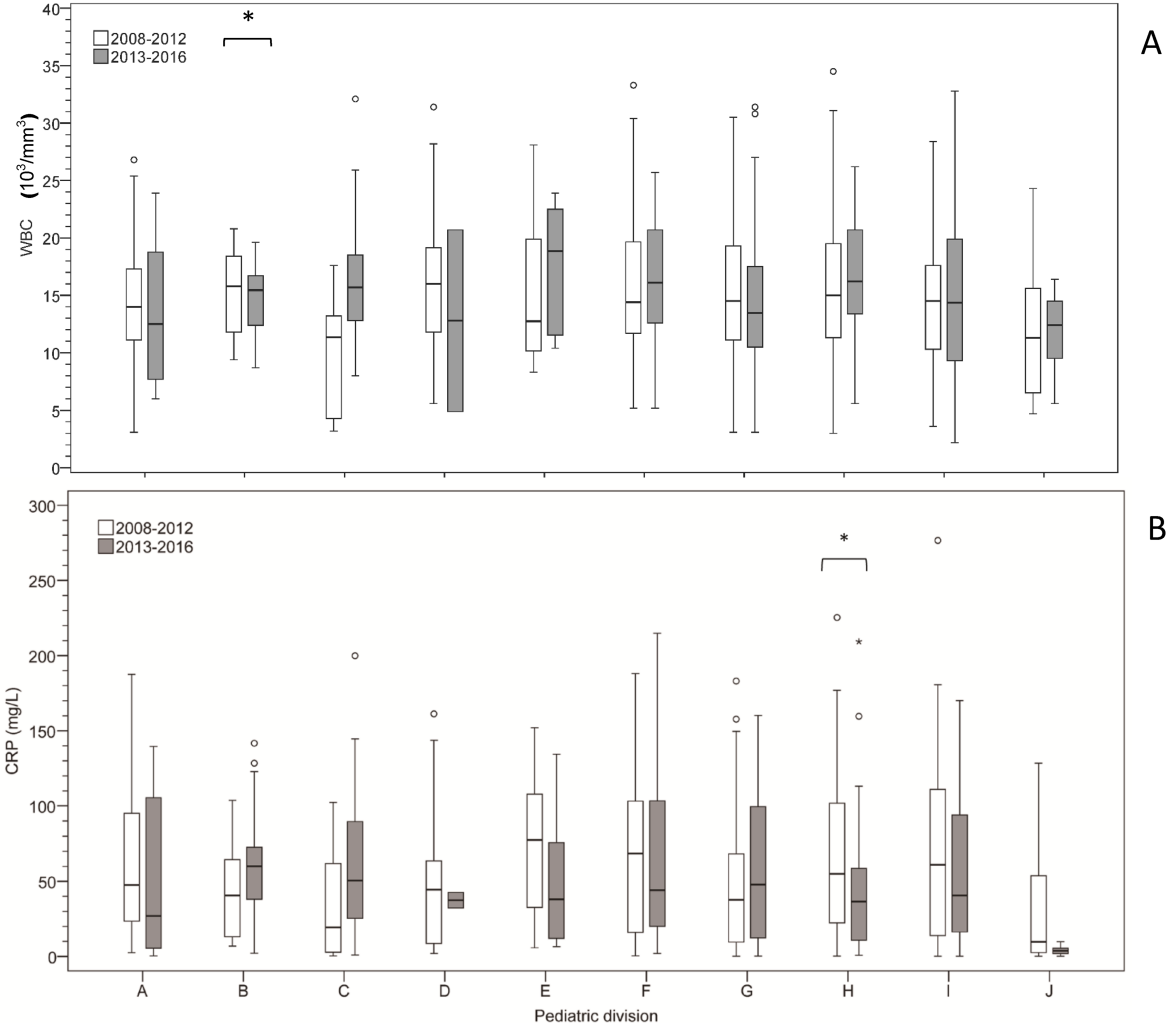
The CRP level associated with antibiotics utilization between 10 divisions of Pediatrics in two periods.

The demographic characteristics of patients during these two periods were compared in Table 5 and Fig 2. The proportion of antibiotics prescription was different significantly between divisions of pediatric department not only during the first period (p<0.001) but also the second period (p<0.001). In the analysis of subgroup regarding to antibiotics utilization between the two periods, both CRP level and WBC count were not different significantly. However, the CRP level associated with antibiotics utilization was decreased significantly only in the pediatric division “H” during the second period, compared with the first period (Fig 3).

**Table 5:** The clinical characteristics and demographics of patients during two periods.

Period	Year 2008~2012 (N = 3445)	Year 2013~2016 (N = 2247)	p Value
**Age (years)**^a^	2.1 (1.2 to 3.5)	1.8 (1.1 to 3.1)	<0.001*
**Sex (M/F)**	1989/1456	1286/961	0.707
**Weight of CMI**^a^	0.252 (0.251 to 0.285)	0.273 (0.258 to 0.292)	<0.001*
**Hospital stay (days)**^a^	3 (3 to 4)	3 (3 to 4)	0.156
**Antibiotics utilization**	479 (14%)	271 (12%)	0.044
CRP (mg/L)^a^	50.1 (12.5 to 96.0)	42.5 (11.9 to 84.5)	0.477
WBC (10^3^ /mm^3^)^a^	14.1 (10.9 to 18.6)	15.3 (11.0 to 19.5)	0.200

^a^: median, IQR.

*: by Mann-Whitney test, significant statistically (p<0.001).

## Discussion

Based on the experience of pediatric enteroviral disease healthcare in our hospital, we report that the utilization of antibiotics (12~15%) for these patients is a clinical reality but it is mostly inappropriate[5]. Since antibiotics are not clinically indicated for these patients, such use should be forbidden or limited. Furthermore, our study indicated that both leukocytosis and elevated CRP level played important roles in the motivation to prescribe antibiotics by their doctors. These findings implicated that pediatricians at the hospital depend on these 2 laboratory tests, especially serum CRP level, to determine a medical course of action. If the cut-off level of serum CRP were to be more than 30 mg/L in our cases (Fig 1), the rate of prescribing antibiotics would increase significantly, possibly due to the concern of the pediatrician that the patient had a community-acquired bacterial infection.

However, antibiotics may not be clinically indicated in the medical care of enteroviral infectious disease. Our previous study showed that antibiotics did not shorten the clinical course of enterovirus and resulted in longer hospital stays for patients with high CRP levels (>80 mg/L). The study also revealed secondary bacterial infection seldom occurred in these patients, although some experts recommend antibiotics for oral mucositis [5]. In Table 2, the factors associated with disease severity, except the higher CRP levels, were positively correlated with longer hospital stay. Higher CMI, admission to ICU, use of IVIG were the main risk factors for longer hospital stay; this may be due to patients with severe disease requiring additional, intensive, supportive care to treat sequelae, such as neurologic complications. These findings indicated patients with higher CRP level might not be associated to the severity of disease on admission, which were in concordance with previous studies[15, 16]. In the literature review, previous studies found that a small proportion of cases had elevated serum CRP in the presence of coxsackievirus infection [1, 2, 17]. However, the present case-control study revealed that prescription of antibiotics was not associated with the epidemic year. The study findings suggest that the species of enterovirus might not significantly be associated with pediatricians’ antibiotic prescription behavior.

Previous studies suggested using routine cerebrospinal fluid enterovirus polymerase chain reaction testing (PCR) reduces length of hospitalization and duration of antibiotic therapy in children [18, 19]. However, enteroviral PCR is not available in each hospital and the lumbar puncture is an invasive and painful procedure for both children and their parents. Previous study of enterovirus 71 by Chang LY et al. revealed leukocytosis was a risk factor of serious neurologic complications[16, 20]. In this study, more leukocytosis might be associated with disease severity (p = 0.001), but elevated CRP level was negatively correlated with the length of hospital stay (Table 2). Therefore, neurologic complications might not be associated with elevated CRP level. Furthermore, we presumed that routine cerebrospinal fluid study is not necessary for patients because our previous publication showed no evidence of meningitis in any of our patients with high CRP level [5].

Without clinical indication, the use of antibiotics would increase the expenses of healthcare insurance, which mainly paid for the cost of the drug and the longer length of hospital stay [5, 18, 19, 21]. These results were also noted and showed concordantly in our study, but their inducing factors were not the same. In cases of longer hospital stay for bacterial infectious diseases, inappropriate antibiotic prescribing may result in greater morbidity and have adverse effects on patients. The impact of inappropriate antibiotic prescribing differs and is more complicated in cases of enteroviral disease. In this study, decision-making between different pediatric divisions for such viral infectious diseases was not consistent regarding prescribing of antibiotics for similar patient conditions (Table 4 and Fig 2). These findings indicated that pediatricians had different therapeutic judgement on patients with similar conditions. The proportion of antibiotics utilization during year 2008~2012 and year 2013~2016 were respectively 14% and 12%, which were not significantly different. Therefore, even though the finding of our previous study, inappropriate antibiotics was still used in these patients consequently. In Fig 3, the behavior of prescribing antibiotics associated with CRP level and WBC count in each pediatric division was still similar between the first period and the second period. However, the CRP level during the second period was significantly lower than the first period in the pediatric division “H”. Higher WBC count was acceptable by doctors only in pediatric division “C”. This might result from differences in pediatricians’ attitude and knowledge about antibiotics use, previous medical education, and clinical confidence[9–11]. It is our limitation of this retrospective study to know these by interviewing these pediatricians. Further study and analysis are needed in the future to investigate these possible intrinsic influencing factors.

Previous cohort studies and meta-analyses have revealed that early-life exposure to antibiotics was associated with development of atopic diseases, such as asthma and eczema [22–25]. They also found that early antibiotic exposure during infancy exhibited a dose-dependent relationship to allergy diseases in late adolescence. In our data, the mean age of patient was less than 3 years old. We should be more prudent in using antimicrobial agents for toddlers, although the cost of antibiotic consumption did not present a substantial economic burden in this study.

As the results of this study, antibiotic use according to elevated CRP level and WBC count should not be indicated for patients with enterovirus. Moreover, in the Fig 3, we found the dependence on CRP level by some specialist was worsen recently. The prescription of antibiotics was mostly dependent on the preference and knowledge of the clinician. We conclude that better appraisal of serum CRP level would result in better quality healthcare for patients experiencing enterovirus infection, and possibly prevent serious neurologic complications [17, 18, 26, 27]. Concomitantly, the risk of promoting antimicrobial resistant bacteria and long-term health impacts of antibiotic administration would be decreased.

As our study was designed retrospectively and patients were collected from a single medical center, there are limitations, including bias in medical record reviewing. In addition, our limitation of this study was that we could not interview pediatricians to exactly know intrinsic factors such as their attitudes and background for antibiotics prescribing. Furthermore, the pathogenic mechanism of serum CRP elevation is not understood in this type of infection. Depending on the type of laboratory tests used to detect bacterial infections, findings may be unreliable and presence of infection is often overestimated. In the future, we hope to better understand the factors influencing the antibiotic prescribing behavior of pediatricians by designing and administering questionnaires. The present study provides insight into the disadvantage of over-reliance on CRP levels in prescribing antibiotics in cases of enteroviral infectious diseases among pediatricians.

## Conclusions

This study indicated that antibiotic prescribing was more complex in infectious diseases caused by viruses than in infectious bacterial diseases. Different specialistics of pediatrians significantly had different decision-making for antibiotics prescription in this kind of viral diseases. Elevated CRP serum level was strongly associated with prescription of antibiotics for the healthcare of children experiencing enteroviral disease. The pediatricians of our hospital may depend substantially on CRP level in making the decision to prescribe antibiotics; however, such prescriptions may be inappropriate and unnecessary according to the literature[5]. Therefore, since this study had better understanding the behavior of pediatricians, our results could help colleagues to improve the quality of medical care for these patients in the future.

## Supporting information

S1 File All data from the database of medical and laboratory record in Kaohsiung Chang Gung Memorial Hospital. (XLSX)

S2 File The cost of antibiotics for our patients. (PDF)
