# Supplementary material for: Understanding physician antibiotic prescribing behavior for children with enterovirus infection
Source: PLoS One. 2018 Sep 7;13(9):e0202316. doi: 10.1371/journal.pone.0202316 (PMC6128467; doi:10.1371/journal.pone.0202316)
Supplement: S2 File — (PDF) [file pone.0202316.s002.pdf]

DRUGCLASS	NEWDRUG	SUM_of_SUM_of_SUM_of_NHIAMT
1' CEPHALOSPORIN	Cefazolin	17488.80
1' CEPHALOSPORIN	LIKODIN	64.86
2' CEPHALOSPORIN	Cefuroxime	2878.00
3' CEPHALOSPORIN	CEFTRIAXONE	38474.00
3' CEPHALOSPORIN	Cefixime	24.40
3' CEPHALOSPORIN	Cefotaxime	4270.00
3' CEPHALOSPORIN	Flomoxef	26180.00
3' CEPHALOSPORIN	SEFTEM	228.80
AMINOGLYCOSIDE	Amikacin	355.30
AMINOGLYCOSIDE	Gentamicin	2178.10
ANTIFUNGAL	Fluconazole	0.00
ANTIFUNGAL	NYSTATIN	11.50
GLYCOPEPTIDE	Teicoplanin	7233.00
GLYCOPEPTIDE	Vancomycin	678.00
MACROLIDE	Azithromycin	138.60
NITROIMIDAZOLE	Metronidazole	27.30
PENICILLIN	Amoxycillin	71.00
PENICILLIN	Ampicillin	9718.14
PENICILLIN	Oxacillin	792.00
PENICILLIN	Penicillin g	2873.40
Penicillin/β lactamase inhibitor	AUGMENTIN	116061.00
Penicillin/β lactamase inhibitor	Ampicillin	4543.00
SULFONAMIDE	Cotrimoxazole	4.00
		234293.20
		7809.773333

1"cefa	17553.66
2"cefa	2878
3"cefa	69177.2
aminoglycoside	2533.4
antifungal	11.5
glycopeptide	7911
macrolide	138.6
metronidazole	27.3
aminopenicillin	9789.14
penicillin	2873.4
augmentin	120604
cotrimoxazole	4
oxaciilin	792

NT
USD
